# Utilization of simultaneous saccharification and fermentation residues as feedstock for lipid accumulation in *Rhodococcus opacus*

**DOI:** 10.1186/s13568-017-0484-0

**Published:** 2017-09-29

**Authors:** Rosemary K. Le, Parthapratim Das, Kristina M. Mahan, Seth A. Anderson, Tyrone Wells, Joshua S. Yuan, Arthur J. Ragauskas

**Affiliations:** 1 Department of Chemical & Biomolecular Engineering, University of Tennessee, Knoxville, TN 37996 USA; 2 Center for Renewable Carbon, Department of Forestry, Wildlife, and Fisheries, University Tennessee Institute of Agriculture, Knoxville, TN 37996 USA; 3 Joint Institute for Biological Sciences, Biosciences Division, Oak Ridge National Laboratory, Oak Ridge, TN 37830 USA; 4 Department of Plant Pathology & Microbiology, Texas A&M University, College Station, TX 77843 USA

**Keywords:** Dilute-acid pretreatment, Simultaneous saccharification and fermentation, *Rhodococcus opacus*, Biofuel, Lipids

## Abstract

Use of oleaginous microorganisms as “micro-factories” for accumulation of single cell oils for biofuel production has increased significantly to mitigate growing energy demands, resulting in efforts to upgrade industrial waste, such as second-generation lignocellulosic residues, into potential feedstocks. Dilute-acid pretreatment (DAP) is commonly used to alter the physicochemical properties of lignocellulosic materials and is typically coupled with simultaneous saccharification and fermentation (SSF) for conversion of sugars into ethanol. The resulting DAP residues are usually processed as a waste stream, e.g. burned for power, but this provides minimal value. Alternatively, these wastes can be utilized as feedstock to generate lipids, which can be converted to biofuel. DAP-SSF residues were generated from pine, poplar, and switchgrass. High performance liquid chromatography revealed less than 0.13% monomeric sugars in the dry residue. Fourier transform infrared spectroscopy was indicative of the presence of lignin and polysaccharides. Gel permeation chromatography suggested the bacterial strains preferred molecules with molecular weight ~ 400–500 g/mol. DAP-SSF residues were used as the sole carbon source for lipid production by *Rhodococcus opacus* DSM 1069 and PD630 in batch fermentations. Depending on the strain of *Rhodococcus* employed, 9–11 lipids for PD630 and DSM 1069 were observed, at a final concentration of ~ 15 mg/L fatty acid methyl esters (FAME) detected. Though the DAP-SSF substrate resulted in low FAME titers, novel analysis of solid-state fermentations was investigated, which determined that DAP-SSF residues could be a viable feedstock for lipid generation.

**Electronic supplementary material:**

The online version of this article (doi:10.1186/s13568-017-0484-0) contains supplementary material, which is available to authorized users.

## Introduction

Transportation is the fastest growing sector, and the second largest energy consuming sector in the US (28%) after electric power (40%) (US-EIA 2015). Therefore, identifying sustainable, carbon neutral alternatives for liquid transportation fuels is at the forefront in green energy research due to concerns over environmental risks, energy security, and the impending depletion of low-cost petroleum reserves, which have global impacts (Ragauskas et al. 2014). The production of renewable drop-in biofuels has garnered great interest (Beckham et al. 2016; Bond et al. 2014; Brown and Brown 2013; Karatzos et al. 2014; Linger et al. 2014; Mu et al. 2013; Ragauskas et al. 2014; Rye et al. 2010; Vardon et al. 2015). For example, biodiesel is a “drop-in” substitute fuel with combustion properties similar to petroleum diesel and is degradable, renewable, and compatible with current global transportation infrastructure. However, the practical availability of non-food based oils on a volume sufficient to displace more than 10% of petroleum based fuels is not currently possible (Xie et al. 2016). Advancements in biorefining technologies along with the development of novel fermentation strategies and analysis will be paramount in order to establish supplementary and sustainable biofuel pathways.

Second-generation lignocellulosic biomass, which encompasses agricultural residues, energy crops and woody biomass, is widely considered an ideal renewable feedstock for bioconversion processes that ultimately yield advanced biofuels (Wyman et al. 1992). However, conversion of these sources to sugars and other useful organic compounds to fuels is hindered by the physicochemical structure and composition of the lignin–hemicellulose matrix, which can prevent hydrolysis of the cellulose present (Olofsson et al. 2008). A multitude of pretreatments have been implemented to degrade and solubilize the more recalcitrant constituents of lignocellulosic biomass via delignification methods, which have been shown to improve subsequent enzymatic hydrolysis towards bioethanol production. Effective pretreatments of lignocellulosic biomass are those that result in the disruption of the cell wall and alter cellulose crystallinity and association with lignin, thereby enhancing the accessibility of cellulose, which increases the yield of desired products in subsequent microbial metabolism. Some of these methods include: alkali treatment and ammonia explosion, among others, and these techniques have resulted in yields of 90% of the theoretical yield of sugar (Kumar et al. 2009). Dilute acid pretreatment (DAP) followed by simultaneous saccharification and fermentation (SSF) is a commonly employed pretreatment strategy to produce a variety of sugars that can be used for bioethanol production (Brodeur et al. 2011; Saha et al. 2005). Under moderate conditions, dilute acid is sufficient to hydrolyze hemicelluloses, however, hydrolysis of cellulose requires more extreme conditions. In ethanol production, dilute-acid pretreatment is commonly coupled with SSF to convert sugars to ethanol (Won et al. 2012). The use of SSF generally results in higher product yields and requires less enzyme loading, compared to other methods, due to the reduction of end-product inhibition by cellobiose and glucose formed during enzymatic hydrolysis (Olofsson et al. 2008). Utilizing SSF has become an attractive method to convert sugars released from cellulose hydrolysis, due to decrease in cost, by decreasing enzyme loading and utilizing one reaction vessel (Abe and Takagi 1991; South et al. 1995). The released sugars can then be metabolized by microorganisms such as yeast, fungi, or soil bacteria, to yield ethanol. Interestingly, the residual fractions and waste effluents generated after the fermentation of sugars could potentially be processed by aromatic degrading oleaginous organisms towards the production of biodiesel, in an optimization step that could profoundly improve biorefining operations.

The use of oleaginous bacteria to accumulate lipids for biofuel production from industrial waste is a relatively novel area of biotechnology and is being studied extensively (Chen et al. 2009; Gomez et al. 2016; Leiva-Candia et al. 2014; Munch et al. 2015; Yousuf 2012; Yu et al. 2011; Zhou et al. 2013). Though engineered algae and yeast are prominent in the field, the oleaginous soil bacteria in the family *Rhodococcus* is an emerging candidate for study due to a broader threshold of suitable nutrients and cultivation conditions (Alvarez et al. 2000; Gomez et al. 2016; Salvachúa et al. 2015). In recent years, *Rhodococcus opacus* (*R. opacus*) strains DSM 1069 and PD630 have been investigated for their ability to accumulate significant amounts of intracellular single cell oils (Kosa and Ragauskas 2011, 2012, 2013; Wei et al. 2015a, b, c). For example, *R. opacus* PD630 has been shown to exhibit oleaginicity ~ 80% on a cell dry weight basis (CDW) when glucose was utilized as a carbon source under nitrogen-limited conditions (Alvarez et al. 2000; Alvarez and Steinbuechel 2002). *Rhodococcus* has also demonstrated the capability to metabolize and degrade aromatic compounds found in lignocellulosic biomass utilizing the β-ketoadipate pathway (Wells and Ragauskas 2012). For example, *R. opacus* PD630 and DSM 1069 have been shown to utilize Kraft lignin as a sole source of carbon and energy to accumulate ~ 5% CDW of cell dry weight in lipids, and later simultaneously utilized aromatic and carbohydrate compounds in pine organosolv pretreatment effluents to accumulate ~ 26% CDW in lipids (Kosa and Ragauskas 2013; Wells et al. 2015).

In this study, the residues from a dilute-acid pretreatment coupled with simultaneous saccharification and fermentation (DAP-SSF) of pine, poplar, and switchgrass were used as the sole resources of carbon and energy for the cultivation of oleaginous *R. opacus* DSM 1069 and PD630. Due to the majority of the residues being solids, it was necessary to perform fermentation with both strains in a suspension of the DAP-SSF residue. Currently, solid-state fermentations with bacteria are generally avoided due to irreversible adsorption of cells to solid materials, thereby presenting a substantial challenge with regard to cell growth characterization. In the work presented here, novel strategies for characterizing the slurried residues to evaluate them as a viable feedstock for cell growth and lipid production were implemented. Cell growth during solid-state fermentations relied on determining the living cell concentration (colony forming units/mL, CFU/mL). The effluent substrate was characterized with respect to the remaining portion of monosaccharides via high-performance anion exchange chromatography with pulsed amperometric detection (HPAEC-PAD) and the determination of the molecular weight of solubilized aromatic components via gel permeation chromatography with UV detection (GPC). FTIR was used to determine structural changes to the lignin residues throughout the metabolism by the *Rhodococci* strains. The compositions and total content of the accumulated lipids at different periods during fermentation were characterized with gas chromatography–mass spectrometry (GC–MS).

## Materials and methods

### Substrates

Debarked pine (*Pinus taeda*) (from USA pulp mill), poplar, (from USA pulp mill) and Alamo switchgrass (from University of Georgia, Athens, GA) were milled to a 2 mm particle size with a Wiley Mill and extractives were removed by Soxhlet extraction with dichloromethane for 48 h. The extracted biomass samples were air-dried and frozen at − 20 °C until usage.

### Dilute-acid pre-treatment, equipment and process

Dilute-acid pre-treatment was conducted based on work by Saha et al. (2005). The treatment parameters were as follows: water to biomass ratio of 10:1 (w/w dry biomass) and 1.5% H_2_SO_4_ (w/w dry biomass). Samples were heated for approximately 45 min while maintaining a mean temperature of 180 °C. Upon completion, the heater for the Parr reactor was turned off and the pressure was slowly vented to return the vessel to atmospheric pressure and the pretreated biomass was removed from the glass liner and cooled to room temperature before freezing at − 20 °C until use.

### Bacterial strains, media, and cultivation

#### Strains

The D_5_A ethanol-producing strain of *Saccharomyces cerevisiae* (ATCC^®^ 200062™) was obtained from ATCC (https://www.atcc.org/). *Rhodococcus opacus* (DSMZ 1069, hereby referred to as DSM 1069) and *R. opacus* PD630 (DSMZ 44193, hereby referred to as PD630) were obtained from the German Collection of Microorganisms and Cell Cultures (DSMZ, http://www.dsmz.de).

#### Media

All media components were obtained from Fisher Scientific (Hampton, NH, USA) or Sigma Aldrich (St. Louis, MO, USA) and used as received. The D_5_A yeast strain was initially cultivated in YP media amended with 5% w/v final glucose as described by Dowe and McMillan (NREL/TP-510-42630) (Dowe and McMillan 2008). During simultaneous saccharification and fermentation (SSF) the yeast was cultured in 7.5% w/w cellulose, 1% w/v yeast extract, 2% w/v peptone, 0.05 M citrate buffer (pH 4.8), 15 units cellulase enzyme from *Trichoderma reesei* (Sigma C2730), per gram cellulose and 50 units beta-glucosidase from almond (Sigma G4511) in 100 mL media.

Shake flask fermentations were carried out with two types of media for each of the *Rhodococci* strains: a full media and a minimal media, which were composed of different carbon and nitrogen sources. The full media was prepared according the formulations as recommended by DSMZ: Medium 65, GYM Streptomyces Medium for DSM 1069 and Medium 535, Trypticase Soy Broth medium for PD630. The minimal media for DSM 1069 was composed of the following chemicals: 0.40 g KH_2_PO_4_, 1.60 g K_2_HPO_4_, 0.20 g MgSO_4_·7H_2_O, 0.03 g FeCl_3_, 0.50 mg MnSO_4_·H_2_O, 1.00 mg CuSO_4_·5H_2_O, 1.00 mg ZnSO_4_·7H_2_O, 0.50 mg CaCl_2_, 0.10 mg KCl, and 0.50 mg H_3_BO_3_/L distilled water (Kosa and Ragauskas 2012). For PD630, the minimal media contained: 9.00 g Na_2_HPO_4_·12H_2_O, 1.50 g KH_2_PO_4_, 0.20 g, MgSO_4_·7H_2_O, 1.20 mg FeNH_4_ citrate, 20.00 mg CaCl_2_, 2.00 mL Hoagland solution (Sigma, H2395), and 0.50 g NaHCO_3_/L distilled water (Schlegel et al. 1961). The nitrogen source for minimal media for DSM 1069 was NH_4_NO_3_ and the source for PD630 was NH_4_Cl. During the cell adaptation phase of cell culture, nitrogen was supplemented at 0.1 and 0.05% w/v during lipid accumulation phase.

### Yeast cultivation

Simultaneous saccharification and fermentation of D_5_A yeast using pine, poplar, and switchgrass biomasses as the carbohydrate source were carried out anaerobically at 35 °C, 130 rpm for 160 h as described by Dowe and McMillan (NREL/TP-510-42630) (Dowe and McMillan 2008). Small aliquots (1 mL) were sterilely removed from each flask at 24, 96, and 160 h, in duplicate, and saved for ethanol detection with an EnzyChrom Ethanol Assay Kit (ECET-100). During the sampling times, the fermentation broth was streaked on solid YP media to test for viability and contamination.

### Simultaneous saccharification and fermentation (SSF) treatment

SSF was conducted based on NREL protocol TP-510-42630 (Dowe and McMillan 2008). The yeast implemented was *Saccharomyces cerevisiae* D_5_A (*S. cerevisiae* Meyen ex E.C. Hansen ATCC^®^ 200062TM). The enzyme loading was 15 units/g cellulose, at 7.0 g cellulose/g biomass, 105 units of cellulase enzyme from *Trichoderma reesei* (Sigma Aldrich, C2730-50ML) was loaded into each 100 mL flask, and 50 units of β-glucosidase from almonds was also added. The flasks were incubated at 35 °C, 130 rpm. The ethanol production data (% ethanol, g/L, % cellulose conversion) for 0, 24, 96, and 160 h were also determined as a function of time (in Additional file 1). Post-SSF, the solid residue was separated from the fermentation broth via vacuum filtration using a Büchner funnel fitted with Whatman Grade 1 filter paper (Sigma Aldrich, WHA1001150) and a side-arm flask. The solid portion of the pretreatment residue was washed with 500 mL of water three times to remove residual yeast fermentation broth and was freeze-dried with a VirTis Freezemobile 25 L freeze dryer for 48 h.

### *Rhodococcu*s cultivation

*Rhodococcus* strains were incubated at 28 °C, 150 rpm with 1.5% (w/v) freeze-dried DAP-SSF residue (2.25 g/150 mL) loading in minimal media. The strains underwent initial adaptation on the residues at 1.5% solids in 10 mL of 0.1% w/v nitrogen minimal media for 24 h. After the initial adaptation phase, all cells and residues were transferred to 150 mL of 0.05% w/v nitrogen minimal media and 1.5% solids loading, that was sterilized by autoclaving for 20 min at 121 °C. The flasks were incubated for 96 h, with sampling at 0, 12, 24, 48, 72, and 96 h. At each sampling time point, the insoluble solid fraction of the fermentation broth was separated from the liquid media and saved for analysis as described above.

### Serial dilution and plating/colony count

Due to the solid-state nature of the biomass/feedstock, optical density (OD) could not be utilized to monitor cell growth as a function of time. Instead, serial dilution and plating was used to track cell viability at 0, 12, 24, 48, 72, and 96 h. For each strain/biomass, 1 mL was removed sterilely from the culture flask and diluted in 9 mL of sterile physiological salt solution, and repeated to dilutions of 10^−2^, 10^−3^, 10^−4^, and 10^−6^. A sample of each dilution, 100 μL, was plated on full media agar for the respective *Rhodococci* strains. The plates were incubated at 28 °C overnight and colonies were counted.

#### Sugar release and quantification of monosaccharides using high performance anion exchange chromatography with pulsed amperometric detection (HPAEC-PAD)

Quantification of monosaccharides in the respective fermentation broths were performed using high performance anion exchange chromatography with pulsed amperometric detection (HPAEC-PAD). The soluble/liquid portion, removed directly from the fermentation broth at 0 and 96 h, and freeze-dried substrate from *Rhodococcus* fermentation of DAP-SSF pretreated residues after acid hydrolysis were analyzed. For the latter preparation, the freeze-dried residues of the initial starting material (0 h) and at the end of the fermentation (96 h) was treated with a two-step H_2_SO_4_ hydrolysis following NREL/TP-510-42618 (Sluiter et al. 2008). In the first step, the DAP-SSF residues were hydrolyzed with 72% (w/w) H_2_SO_4_ at 30 °C for 1 h. In the second step, the hydrolyzed samples were diluted to 3% H_2_SO_4_ (w/w) of final concentration, followed by autoclaving at 121 °C for 1 h. The hydrolysate was filtered from solid residue and the recovered liquid fraction was analyzed with a Dionex ICS-3000 ion chromatography system (Thermo Fisher Scientific, Sunnyvale, CA) to determine the sugar profile of the fermentation residues. Specifically, a Dionex ICS-3000 ion chromatography with CarboPac PA-1 column was utilized. The temperature of the column was set to 30 °C and the eluents A and B were 100% DI water and 200 mM NaOH, respectively. The flow rate was set to 0.35 mL/min. Duplicate samples of the fermentation broths were analyzed and each analyte was quantified using a standard curve based on calibration curves of an external standard of glucose, xylose, mannose, galactose, arabinose.

### Gel permeation chromatography (GPC)

The DAP-SSF residues, before and after exposure to *Rhodococcus*, and after vacuum filtration as previously described above, were dried under vacuum at 40 °C overnight and then were acetylated with acetic anhydride/pyridine (1/1, v/v) at ambient temperature for 24 h in a sealed flask under an inert atmosphere. The concentration of the lignin in the solution was approximately 20 mg/mL. After 24 h, the solutions were diluted with ~ 20 mL of ethanol and stirred for an additional 30 min, after which the solvents were removed with a rotary evaporator, followed by drying in a vacuum oven at 40 °C. Prior to GPC analysis the acetylated pre- and post-*Rhodococcus* fermentation residue samples were dissolved in tetrahydrofuran (1.0 mg/mL), filtered through a 0.45 µm filter, and placed in a 2 mL auto-sampler vial. The molecular weight distributions of the acetylated lignin samples were then analyzed on an Agilent GPC SECurity 1200 system equipped with four Waters Styragel columns (HR1, HR2, HR4, HR6), an Agilent refractive index (RI) detector, and an Agilent UV detector (270 nm), using tetrahydrofuran (THF) as the mobile phase (1.0 mL/min), with an injection volume of 20 μL. A standard polystyrene sample was used for calibration. The number-average molecular weight (M_n_) and weight-average molecular weight (M_w_) were determined by GPC.

### Fourier transform infrared spectroscopy (FTIR)

A PerkinElmer Spectrum 100 FTIR spectrometer with a universal attenuated total reflectance (ATR) sampling accessory (Perkin-Elmer Inc., Wellesley, MA, United States) was used to monitor the structural changes in the residues. The residue samples were pressed uniformly against the crystal surface via a spring-anvil, and the spectra were obtained by 32 scans accumulation from 4000 to 500 cm^−1^ at 4 cm^−1^ resolution. The ATR correction and the baseline correction were carried out using Perkin-Elmer Spectrum software (Perkin-Elmer Inc., Norwalk, CT, United States). The background correction of the obtained spectra was performed using the Spectrum program from Perkin-Elmer.

### Lipid characterization

For methanolysis/transesterification, approximately 15–20 mg of freeze dried cells/residual biomass, for each substrate at several time points, was dissolved in 1.00 mL CHCl_3_, 0.85 mL methanol and 0.15 mL concentrated H_2_SO_4_. The samples were incubated at 100 °C for 140 min in a sandbath to obtain fatty acid methyl esters (FAME) from all lipids present. Subsequently, 0.50 mL of distilled water was added and the samples were shaken vigorously (vortexed) for 1 min, and after phase separation, the FAME-containing organic phase was removed. Approximately 0.6 mL containing the FAME was recovered. Each sample was diluted to 2 mL with chloroform (1:3.33 dilution) before analysis.

GC–MS was used to identify FAME produced by *Rhodococcus* DSM 1069 and PD630, which was performed on an Agilent 7890A GC/5975C MSD system on a HP-5MS column, with 1 μL sample injection. The sample program was run as follows: 0.5 min equilibration time, 50 °C for 2 min, 15 °C/min to 200 °C then held for 5 min, 10 °C/min to 250 °C then held for 2 min, and 25 °C/min to 300 °C then held for 4 min. The Supelco^®^ 37 Component FAME Mix (Sigma-Aldrich CRM47885) was used as an external standard.

AMDIS software by NIST was used to determine the integrated areas of peaks identified as “methyl esters” by the NIST Chemical Library. The integrated areas of each identified peak with 90% or greater compound m/z spectrum similarity of the external standard at five dilutions (undiluted, 1:2, 1:10, 1:20, and 1:40, wt%) were used to generate standard curves for each component. The integrated areas of peaks for identified fatty acid methyl esters at each sampling time point for the pine, poplar, and switchgrass samples were used to approximate the total lipid content (mg/L) at each time point.

## Results

### Growth of *R. opacus* DSM 1069 and PD630 on DAP-SSF residues

**Fig. 1:**
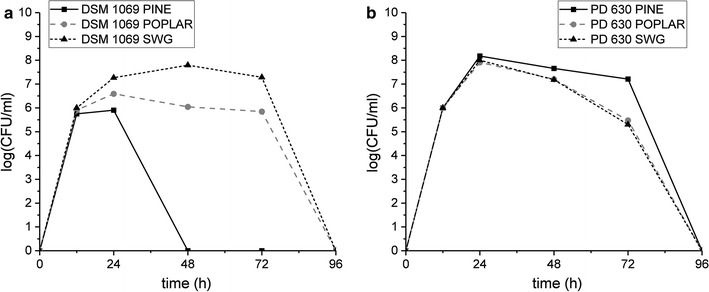
Viability of **a** *Rhodococcus opacus* DSM 1069 and **b** *Rhodococcus opacus* PD630 grown on DAP-SSF pine, poplar, and switchgrass residues over 96 h, determined by serial dilution. The viability of pine is shown with a black solid line and square symbol, poplar with gray, dashed line and circular symbol, and switchgrass with black, small dashed line and triangular symbol

Fermentations of the DAP-SSF residues with *Rhodococci* were performed by suspending 2.25 g of freeze-dried post-DAP-SSF residues into 150 mL of strain-specific minimal media. Cell viability for *R. opacus* DSM 1069 and PD630 grown on DAP-SSF pine, poplar, and switchgrass residues was monitored by tracking colony forming units per volume (CFU/mL) via plating of serial dilutions of the cultures (Kosa and Ragauskas 2013). The results of serial dilutions and plating indicated that the majority of *Rhodococci* strains were able to grow on the residues as their sole carbon source throughout the experiments, as seen in Fig. 1a, b. From 12 to 24 h, the magnitude of CFU/mL universally increased 100-fold. After 24 h of incubation, all strains exhibited CFU/mL magnitudes greater than 10^5^. From 24 to 72 h, the viability of cells largely remained consistent except in the case of DSM 1069 grown in DAP-SSF residues derived from pine. After 72 h, all cells ceased to grow.

### Sugar analysis of DAP-SSF residues

The detection of monosaccharides in the filtered suspension of the DAP-SSF residues was performed using HPAEC-PAD via a Dionex ICS-3000 ion chromatography unit based on previous literature (Hu et al. 2011; Kosa and Ragauskas 2013; Sluiter et al. 2008). The results showed that of the three initial feedstocks, only the pine filtrates had trace and detectable amounts of glucose (9.22 × 10^−3^ mg/mL) and no other monosaccharides. Less than 0.13% monomeric sugars/g dry biomass were detected. By contrast, after acid hydrolysis of the solid residues, the pine contained ~ 1 mg/mL glucose (0.05 g glucose/g biomass) before fermentation with less than 10% reduction and after 96 h of fermentation by *Rhodococcus*, poplar contained ~ 1.8 mg/mL glucose (0.12 g glucose/g biomass) before fermentation with ~ 20% reduction and after 96 h of fermentation by *Rhodococcus*, and switchgrass contained ~ 1.6 mg/mL glucose (0.10 g glucose/g biomass) before fermentation with ~ 1 and 34% reduction after 96 h of fermentation by *Rhodococcus* DSM 1069 and PD630, respectively (shown in Additional file 2: Figure S1).

### Determination of molecular weight distribution of DAP-SSF residues via gel permeation chromatography (GPC)

**Fig. 2:**
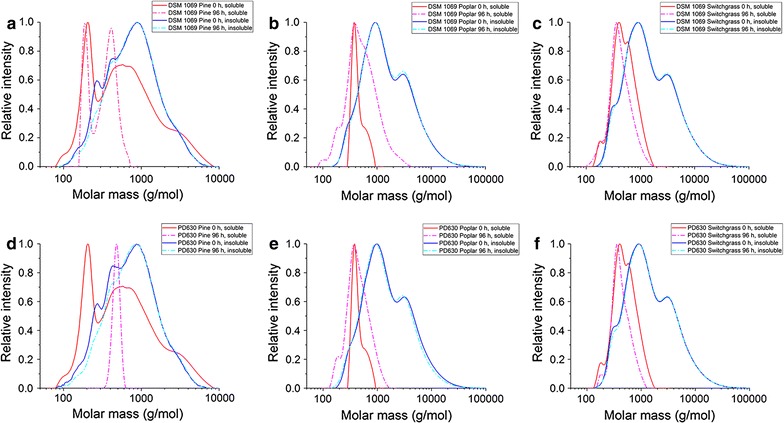
Relative intensity of molar mass (g/mol) distribution of soluble and insoluble DAP-SSF residues from gel permeation chromatography. Distribution of **a** pine residue at 0 and 96 h of DSM 1069 fermentation, **b** poplar residue at 0 and 96 h of DSM 1069 fermentation, **c** switchgrass residue at 0 and 96 h of DSM 1069 fermentation, **d** pine residue at 0 and 96 h of PD630 fermentation, **e** poplar residue at 0 and 96 h of PD630 fermentation, and **f** switchgrass residue at 0 and 96 h of PD630 fermentation. The solid red and dashed magenta lines refer to the soluble fraction at 0 and 96 h of fermentation, respectively, and the solid blue and dashed cyan lines refer to the insoluble fraction at 0 and 96 h of fermentation, respectively

The insoluble/solids and solubilized filtrate suspension fractions of the DAP-SSF residues were analyzed with GPC before and after it was used as the carbon source for *R. opacus* DSM 1069 and PD630 strains, summarized in Fig. 2a–f. Analysis of the insoluble/solid residues showed no significant changes in the molecular weight (< 10% over the course of 96 h of incubation). For both DSM 1069 and PD630 strains, the change in the molecular weight in the soluble/liquid fraction of the pine residue was significant, 65.2 and 50.3% decrease, respectively, where molecules of ~ 900–1000 g/mol were broken down to ~ 400–500 g/mol.

### Fourier transform infrared spectroscopy (FTIR) characterization of lignocellulosic biomass (pine, poplar and switchgrass) after DAP-SSF treatments and before/after treatment with *R. opacus* PD630 and DSM 1069

FTIR spectroscopy was performed on DAP-SSF pretreatment solid residues before and after fermentation with *Rhodococcus*. Spectra of the starting residues, after DAP and SSF pretreatment, were consistent with reported spectral data for soft wood lignin (guaiacyl type) and similarly for poplar and switchgrass, which consists mainly of S- and G-type lignin (shown in Additional file 3: Figure S2, Additional file 4: Figure S3) (Kline et al. 2010; Meng et al. 2016; Xu et al. 2013). Furthermore, the resulting spectra for the residues after *Rhodococcus* treatment indicated they were consisted largely of corresponding lignin signals, but the bulk of the signal remained unchanged after fermentation.

### Characterization of lipids produced by *Rhodococcus* using DAP-SSF residues as a feedstock

**Fig. 3:**
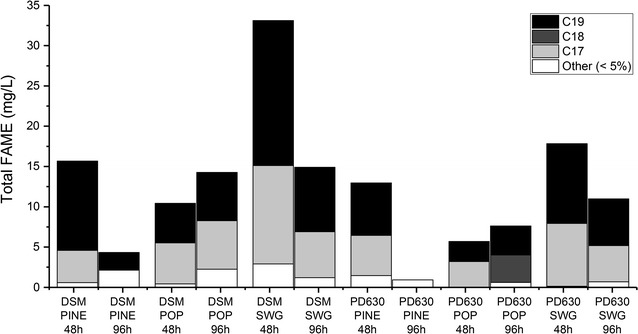
Distribution of total FAME by carbon chain length produced by *R. opacus* DSM 1069 and PD630 grown on pine, poplar, and switchgrass residues at 48 and 96 h. The black bars indicate the total FAME of 19-carbon length, dark gray—total FAME of 18-carbon length, light gray—total FAME of 17-carbon length, and white—all other FAME contributing to less than 5% of the total

Each of the DAP-SSF residues generated lipids, which were converted to FAME by acidic methanolysis. For both strains, DSM 1069 and PD630, the switchgrass DAP-SSF residue performed as the best substrate for overall sustained growth and lipid production over time. Both DSM 1069 and PD630 accumulated the most FAME on switchgrass residue after 48 h, 33.1 mg/L and 17.7 mg/L, respectively. The majority of the lipid accumulation occurred after the exponential growth phase at 48 h and was not maintained over time, decreasing by 50% or more when the cells were grown on pine and switchgrass residues. However, when both DSM 1069 and PD630 were grown on poplar residue, the maximum FAME was detected at 96 h. Figure 3 summarizes the carbon chain distribution of the total FAME produced (mg FAME/L) by *R. opacus* DSM 1069 and PD630.

When DSM 1069 strain was grown on pine, poplar, and switchgrass residues, all samples showed the presence of lipids as detected by conversion to FAME. At 48 h, the pine residue initially contained branched tetradecanoic acid, and hexadecanoic/hexadecenoic acids (C17), and octadecanoic acid (C19) methyl esters, detected at 25.5% C17 and 70.8% C19 length FAME, with ~ 1% each of C5, C13, and C14 FAME. At 96 h, 50% of the FAME detected was from C19, 29.5% C17, and the remainder was contributed by C11, C13, and C18, contributing less than 10% each. For poplar, at 48 h, the cells generated hexadecenoic acid, with the percentage of C17 FAME contributing to 49.0% of the total and C19 octadecanoic acid FAME at 47.0%. By 96 h, the C17 and C19 FAME decreased by 5–7%, giving rise to C11 (1.2%), C13 (2.2%), and 8.9% C18. The switchgrass residue resulted in the detection of predominately hexadecanoic/hexadecenoic acid (C17) and octadecanoic/octadecenoic acid (C19) methyl esters at 36.7 and 54.5%, respectively, with minimal contributions C13 (dodecanoic/dodecenoic), C15 (tetradecanoic, tridecanoic), C16 (branched tetradecanoic), and C18 (heptadecanoic) FAME of 2.5% or less. At 96 h, the distribution of the FAME remained relatively the same, with C17 and C19 FAME dominating the distribution at 38.3 and 53.6%, respectively. However, nonanoic acid methyl ester (C10) appeared and was detected at 4.9% of the total.

PD630 growth on the residues resulted in similar FAME detected. Pine yielded a variety of FAME: C13 (dodecanoic/dodecenoic) at 1.7%, C14 (tridecanoic) at 1.3%, C17 (hexadecanoic/hexadecenoic, branched tetradecanoic) at 25.5%, and C19 (octadecanoic/octadecenoic) contributing to 70.8% of the total FAME produced. At 96 h, the C19 FAME were reduced by 20%, which gave rise to C11 (4.3%), additional C13 (7.8%), and C18 (7.5%) FAME. The poplar residue resulted in C17 (pentadecanoic, hexadecenoic/hexadecanoic) and C19 (octadecanoic/octadecenoic) FAME at 55.4 and 44.6%, respectively at 48 h. The C19 FAME contribution remained about the same at 96 h, and the C17 FAME contribution decreased by ~ 10%, giving rise to 7.4% C8 (hexanedioic) and 0.6% C16 (branched tetradecanoic) FAME. The switchgrass residue resulted branched tetradecanoic acid and hexadecanoic/hexadecenoic acid (C17, 43.7%), and octadecanoic/octadecenoic acid (C19, 55.6%) methyl esters at 48 h. At 96 h, the C17 and C19 FAME titer decreased by ~ 3% each, giving rise to decanoic acid (C11), dodecanoic acid (C13), and tridecanoic acid (C14) methyl esters at ~ 2% each of the total.

## Discussion

The residues from dilute-acid pretreatment coupled with simultaneous saccharification and fermentation (DAP-SSF) of pine, poplar, and switchgrass were used as sole carbon sources for cultivation of oleaginous *R. opacus* DSM 1069 and PD630. Due to the insoluble nature of the DAP-SSF residue feedstocks, it was necessary to perform the fermentations with *Rhodococci* in a suspension of the solids. The results of this slurry cell culture indicated cell growth is viable utilizing DAP-SSF residues as sole sources of carbon and energy to produce single cell oils. The cell viability for *R. opacus* DSM 1069 and PD630 grown on DAP-SSF residues was determined by tracking colony forming units per volume via plating of serial dilutions of culture (Kosa and Ragauskas 2013). This method was utilized as the primary means of tracking cellular growth as opposed to optical density tracking with UV–visible spectroscopy and cell dry weight due to the inability to completely separate cells from compact residues in the suspension broth. Drawbacks of this alternative technique include the extended time for cells to grow in between intervals of measurement. In addition, the lack of cell dry weight prevents the ability to determine cell oleaginicity and final conversion of residues to lipids. However, for all residues, both strains of *R. opacus* were able to sustain growth. The viability of the *Rhodococci* strains on the DAP-SSF residues suggest that the cells survived by metabolizing small dissolved lignin molecules, supported by the GPC results (Fig. 2).

Gel permeation chromatography was used to determine the molecular weight distributions of molecules in the fermentation broths before and after *Rhodococci* treatment, which revealed the molecular weights were significantly different for the soluble/dissolved fraction compared to the insoluble/compact residues. For all of the residues, the cells preferentially degraded the molecules present in the dissolved fraction, where the strains metabolized the molecules to a detectable extent, particularly molecules that had a molecular weight of ~ 400–500 g/mol. Due to higher surface area and lower molecular weight distributions of the soluble fraction, these compounds were likely targeted by cells, over the larger and inaccessible insoluble and compact residues, which is why more significant modifications were observed in solubilized suspension fractions during incubation with *Rhodococcus*. Molecules in the soluble fraction, with a molecular weight of 900–1000 g/mol were broken down to ~ 400–500 g/mol, whereas the insoluble fractions saw changes of less than ~ 10% in the molecular weight. Therefore, these results suggest that there is an optimal molecular weight range that is more favorable for metabolism by these organisms. These results are consistent with previous studies that show low molecular weight lignin sources are important for efficient microbial transformation of lignin to renewable platform compounds (Abdelaziz et al. 2016; Palazzolo and Kurina-Sanz 2016).

Furthermore, the resulting FTIR spectra for the residues after *Rhodococcus* treatment indicated they were consisted largely of corresponding lignin signals, but the bulk of the signal remained unchanged after fermentation. These results were consistent with the hypothesis that *Rhodococcus* is selective for low molecular weight materials and not specific functional groups, as supported by the GPC results of the solid residues.

HPLC did not detect the presence of monomeric sugars after simultaneous saccharification and fermentation of the DAP pretreatment residues and all other monomeric sugars were below detection limits for all other samples and intervals. However, this technique did not account for residual sugars that are not monomeric as shown by the hydrolyzed residue samples with a substantial contribution of glucose, despite a cellulose conversion of ~ 70% during SSF, typically observed for this pretreatment (Dowe and McMillan 2008).

Using GC–MS to detect the accumulated lipids throughout the fermentations resulted in a maximum variety of 9 or 11 compounds, for PD630 and DSM 1069, respectively, for these particular residues. Though the total FAME that were produced was limited to 15–35 mg/L FAME at the maximum detection, the FAME that were produced are of useful chain lengths in the biofuel field. At 96 h, the distribution of the FAME remained relatively the same, with C17 and C19 FAME dominating the distribution at 38.3 and 53.6%, respectively. However, nonanoic acid methyl ester (C10) appeared and was detected at 4.9% of the total. The shorter fatty acids detected in this “declining” phase are potentially semi-digested fatty acids. With the majority of the total lipid titers being contributed by C17 and C19 FAME across all three biomasses and both *Rhodococcus* strains, this suggests that the cells have a preference towards these carbon lengths when grown on DAP-SSF residues. In addition, both strains generated the most FAME when switchgrass pretreated residues were used as the carbon source, ~ 32.5 mg/L FAME for DSM 1069 at 48 h and ~ 18 mg/L FAME for PD630 at 48 h. When poplar was used as the carbon source, both strains maintained relatively the same total FAME content over the course of the fermentation, 10–15 and 6–8 mg/L FAME for DSM 1069 and PD630, respectively. However, when the pine residue was the sole carbon source, DSM 1069 and PD630 initially maintained higher content of FAME, ~ 15 and ~ 13 mg/L, respectively, and depleting to less than 5 mg/L after 96 h. These results suggest a preference for specific lignin moieties, and distinctive utilization based on the biomass used as the carbon source. With both strains generating the most FAME on the switchgrass residues, it suggests a preference for H-type lignin, which grass-type biomass contain a significant amount of (David and Ragauskas 2010) and is more easily utilized compared to G and S-type lignin since they can be more easily metabolized (Kosa and Ragauskas 2012; Le et al. 2017).

Furthermore, these insoluble residues show an improvement over other purified lignin streams, achieving less than 10 mg/L FAME (Kosa and Ragauskas 2013; Wei et al. 2015a). If the strains of *Rhodococci* were engineered to increase titers of these specific and useful lipids (C5-C23) this pre-treatment residue could be a beneficial platform for transportation fuels. Targeting specific particle sizes and optimizing the solid-state fermentation broth could enhance bacterial utilization of residues and result in increased titers or a wider range of useful products.

## Additional files

**Additional file 1:** Summary of ethanol production during DAP-SSF treatment.

**Additional file 2: Figure S1.** Summary of sugars present in DAP-SSF residue before and after fermentation with *Rhodococcus* after acid-hydrolysis.

**Additional file 3: Figure S2.** FTIR spectra of poplar solid residue (obtained after DAP-SSF pretreatment) (a) before and after treatment with two different strains of *R. opacus* for 96 h, (b) zoomed in fingerprint region of the spectra, and (c) comparison of the relative intensity of the peaks at 1512, 1315 and 1108 cm^−1^; each spectrum represented is the average of at least two spectra recorded for each sample.

**Additional file 4: Figure S3.** (a) FTIR spectra of switchgrass residue (obtained after DAP-SSF pretreatment) before and after treatment with two different strains of *R. opacus* for 96 h, (b) zoomed in fingerprint region of the spectra, and (c) comparison of the relative intensity of the peaks at 1513, 1316 and 1107 cm^−1^; each spectrum represented is the average of at least two spectra recorded for each sample.

## Abbreviations

ATR: attenuated total reflectance
CDW: cell dry weight basis
CFU/mL: colony forming units/mL
DAP: dilute-acid pretreatment
DAP-SSF: dilute-acid pretreatment coupled with simultaneous saccharification and fermentation
FAME: fatty acid methyl esters
FTIR: fourier transform infrared spectroscopy
GC–MS: gas chromatography–mass spectrometry
GPC: gel permeation chromatography
HPAEC-PAD: high-performance anion exchange chromatography with pulsed amperometric detection
HPLC: high performance liquid chromatography
OD: optical density
*R. opacus*: *Rhodococcus opacus*
SSF: simultaneous saccharification and fermentation
