# Supplementary material for: Utilization of simultaneous saccharification and fermentation residues as feedstock for lipid accumulation in Rhodococcus opacus
Source: AMB Express. 2017 Sep 29;7:185. doi: 10.1186/s13568-017-0484-0 (PMC5622019; doi:10.1186/s13568-017-0484-0)
Supplement: Supplementary file 1 — Additional file 1: Summary of ethanol production during DAP-SSF treatment. [file 13568_2017_484_MOESM1_ESM.docx]

***AMB Expr***

**Utilization of simultaneous saccharification and fermentation residues as a feedstock for lipid accumulation in *Rhodococcus opacus***

Rosemary K. Le^1^, Parthapratim Das^1^, Kristina M. Mahan^1^, Seth A. Anderson^1^, Tyrone Wells, Jr.^1^, Joshua S. Yuan^4^, Arthur J. Ragauskas^1,2,3*^

**Supplementary data**

***Ethanol detection in SSF broth***

Ethanol detection in the SSF broth for each respective biomass was performed, in duplicate, following the BioAssay Systems protocol for the EnzyChrom Ethanol Assay Kit (ECET-100). Each of the 1 mL aliquots, a mixture of the broth and residue, were spun at 5,000 g in a microcentrifuge for 10 minutes. For each sample, 30 μL of the supernatant was mixed with 270 μL of working reagent and incubated for 30 min at room temperature, after which a reagent was added to stop the reaction. A calibration curve was also prepared, as described by the protocol. If the samples appeared to contain more ethanol by visual inspection, darker in color, than the standard samples, the working samples were diluted 10-fold with water. The optical density of the samples for the calibration curve and the working samples were measured at 565 nm using a Shimadzu (Model no.: UV160U) spectrophotometer. Equation 1 was used to calculate the concentration of ethanol:

$$\text{Eq. 1: }\left[ \text{Ethanol} \right]\text{ = }\frac{\text{ΔOD}_{\text{565}}}{\text{slope}}\text{ × n }\left( \text{\%} \right)$$

where ΔOD_565_ is the difference in the optical density of the sample and the blank at 565 nm, n is the dilution factor (10 for a 1:10 dilution).

The percent cellulose conversion was determined by Equation 2 (NREL/TP-510-42630):

$$\text{Eq. 2: \% cellulose conversion = }\frac{\text{[EtOH]}_{\text{f}} -\text{ }\text{[EtOH]}_{\text{0}}}{\text{0.51 × (}\text{f}\text{ × [biomass] × 1.111)}}\text{ × 100\%}$$

where [EtOH]*_f_* is the final ethanol concentration the end of the fermentation in g/L, [EtOH]*_0_* is the ethanol concentration at the beginning of the fermentation in g/L, which should be zero, [biomass] is the concentration of dry biomass at the beginning of the fermentation in g/L, *f* is the cellulose fraction of dry biomass in g/g, 0.51 is the conversion factor for glucose to ethanol, based on stoichiometry for yeast fermentation, and 1.111 is the conversion factor for cellulose to equivalent glucose.

***DAP-SSF treatment – ethanol production***

DAP and SSF were performed based on previous literature (Dowe and McMillan 2008; Saha et al. 2005). The ethanol yield on the pretreated pine biomass was 14.68 g/l ± 0.31(standard deviation from the mean), 27.82 g/l ± 0.93 on poplar, and 30.01 g/l ± 2.17 on switchgrass after 160 h. The cellulose conversion were 37.04% ± 0.78, 63.47% ± 2.12, and 74.65% ± 5.39 for pine, poplar, and switchgrass, respectively. Both the ethanol yield and cellulose conversion are comparable to previously reported data with ~30 g/l ethanol yield for SSF of DAP biomass (Olofsson et al. 2008). Cellulose conversion at ~70% is also comparable to literature (Dowe and McMillan 2008). The expected pH (~5.0) and plate streaks of samples at 160 h, when the experiment ended indicate there was no contamination and the data from the SSF is valid.

***Klason Lignin Analysis and Acid Soluble Lignin Analysis***

Klason lignin analysis (KLA) was performed on the pine, poplar and switchgrass residues after dilute acid pretreatment, after simultaneous saccharification and fermentation, and after exposure to the *Rhodococci* strains. The post-SSF and post-*Rhodococcus* fermentation insoluble residues were separated from the respective fermentation broths via vacuum filtration using a Büchner funnel fitted with Whatman Grade 1 filter paper (Sigma Aldrich, WHA1001150) and a side-arm flask. The portion captured on the filter was freeze-dried with a VirTis Freezemobile 25L freeze dryer for 48 h and the fermentation broths were saved at 4 °C until analysis. Klason analysis was performed on the insoluble fraction of the fermentation slurry after freeze-drying based on NREL methods (NREL/TP-510-42618) (Sluiter et al. 2008). The results of KLA are shown in Table S1.

***Klason and acid-soluble lignin analysis***

The Klason lignin content of the extractives-free biomasses, prior to DAP and SSF treatments were 29.90%, 21.21%, and 25.33% for loblolly pine, poplar, and Alamo switchgrass, respectively. The Klason lignin content of the biomasses were determined after dilute acid pretreatment (DAP), after simultaneous saccharification and fermentation (SSF) of the DAP, and after 96 hours of fermentation with *Rhodococcus opacus* DSM 1069 and PD630 strains, summarized in Table 1. As observed in the work by Sannigrahi *et al.* the Klason lignin content increases after SSF (Sannigrahi and Ragauskas 2011).

**Table S1** Klason lignin content of dilute acid pretreated (DAP) biomass, post DAP simultaneous saccharification and fermentation treated biomass (DAP-SSF), and post fermentation with *R. opacus* DSM 1069 and PD630 DAP-SSF treated biomass after 96 h

| Sample | Mass (g) | Avg. % MC | Water Content (mg) | % Klason Lignin | % Acid-  Soluble  Lignin | Mass Klason (g) |
| --- | --- | --- | --- | --- | --- | --- |
| DAP Pine | 0.1858 | 3.25 | 6.0 | 46.12% | 0.917% | 0.0829 |
| DAP Poplar | 0.1834 | 1.70 | 3.1 | 36.22% | 0.404% | 0.0653 |
| DAP Switchgrass | 0.1943 | 1.70 | 3.3 | 33.25% | 0.989% | 0.0635 |
| DAP-SSF Pine | 0.2063 | 3.30 | 6.8 | 44.44% | 0.414% | 0.0886 |
| DAP-SSF Poplar | 0.1806 | 1.70 | 3.1 | 37.63% | 0.378% | 0.0668 |
| DAP-SSF Switchgrass | 0.1966 | 1.65 | 3.2 | 41.12% | 0.906% | 0.0795 |
| 96 h DSM 1069-DAP-SSF Pine | 0.2016 | 3.30 | 6.7 | 63.76% | 0.702% | 0.1243 |
| 96 h DSM 1069-DAP-SSF Poplar | 0.1986 | 1.70 | 3.4 | 48.82% | 0.255% | 0.0953 |
| 96 h DSM 1069-DAP-SSF Switchgrass | 0.1974 | 1.65 | 3.3 | 44.86% | 0.687% | 0.0871 |
| 96 h PD630-DAP-SSF Pine | 0.1958 | 3.30 | 6.5 | 56.14% | 0.608% | 0.1063 |
| 96 h PD630-DAP-SSF Poplar | 0.1995 | 1.70 | 3.4 | 48.09% | 0.269% | 0.0943 |
| 96 h PD630-DAP-SSF Switchgrass | 0.1938 | 1.65 | 3.2 | 43.97% | 0.701% | 0.0838 |

*Insert Fig S1-S3*

**Raw data**

**Serial dilution (CFU/ml)**

| **Time (h)** | **DSM1069 Pine** | **DSM1069 Poplar** | **DSM1069 Switchgrass** | **PD630 Pine** | **PD630 Poplar** | **PD630 Switchgrass** |
| --- | --- | --- | --- | --- | --- | --- |
| 0 | 0 | 0 | 0 | 0 | 0 | 0 |
| 12 | 5.8 | 5.9 | 6.0 | 6.0 | 6.0 | 6.0 |
| 24 | 5.9 | 6.6 | 7.3 | 8.2 | 7.9 | 8.0 |
| 48 | 0 | 6.0 | 7.8 | 7.7 | 7.2 | 7.2 |
| 72 | 0 | 5.8 | 7.3 | 7.2 | 5.5 | 5.3 |
| 96 | 0 | 0 | 0 | 0 | 0 | 0 |

**GC-MS FAME (mg/L)**

| **FAME (mg/L)** | **Other**  **( < 5 %)** | **C17** | **C18** | **C19** | **std error**  **< 5%** | **std error C17** | **std error C18** | **std error C19** |
| --- | --- | --- | --- | --- | --- | --- | --- | --- |
| **DSM PINE 48h** | 0.6 | 4.0 |  | 11.1 | 0.03 | 0.20 | 0.00 | 0.56 |
| **DSM PINE 96h** | 2.1 |  |  | 2.2 | 0.11 | 0.00 | 0.00 | 0.11 |
| **DSM POP 48h** | 0.4 | 5.1 |  | 4.9 | 0.02 | 0.25 | 0.00 | 0.24 |
| **DSM POP 96h** | 2.3 | 6.0 |  | 6.0 | 0.11 | 0.30 | 0.00 | 0.30 |
| **DSM SWG 48h** | 2.9 | 12.2 |  | 18.0 | 0.15 | 0.61 | 0.00 | 0.90 |
| **DSM SWG 96h** | 1.2 | 5.7 |  | 8.0 | 0.06 | 0.29 | 0.00 | 0.40 |
| **PD630 PINE 48h** | 1.5 | 5.0 |  | 6.5 | 0.07 | 0.25 | 0.00 | 0.32 |
| **PD630 PINE 96h** | 0.9 |  |  |  | 0.05 | 0.00 | 0.00 | 0.00 |
| **PD630 POP 48h** |  | 3.2 |  | 2.5 | 0.00 | 0.16 | 0.00 | 0.13 |
| **PD630 POP 96h** | 0.6 |  | 3.4 | 3.6 | 0.03 | 0.00 | 0.17 | 0.18 |
| **PD630 SWG 48h** | 0.1 | 7.8 |  | 9.9 | 0.01 | 0.39 | 0.00 | 0.49 |
| **PD630 SWG 96h** | 0.7 | 4.5 |  | 5.8 | 0.03 | 0.23 | 0.00 | 0.29 |

**GC-MS FAME C-chain distribution**

| **FAME (mg/L)** | **5** | **8** | **10** | **11** | **12** | **13** | **14** | **15** | **16** | **17** | **18** | **19** | **23** | **TOTAL** | **Other**  **( < 5 %)** |
| --- | --- | --- | --- | --- | --- | --- | --- | --- | --- | --- | --- | --- | --- | --- | --- |
| **DSM PINE 48h** | 0.1 |  |  |  |  | 0.3 | 0.2 |  |  | 4.0 |  | 11.1 |  | 15.7 | 0.6 |
| **DSM PINE 96h** |  |  |  | 0.2 |  | 0.3 |  |  |  | 1.3 | 0.3 | 2.2 |  | 4.3 | 2.1 |
| **PD630 PINE 48h** |  |  |  |  |  | 0.6 | 0.3 |  | 0.1 | 5.0 |  | 6.5 | 0.4 | 12.9 | 1.5 |
| **PD630 PINE 96h** |  |  |  |  |  |  |  |  |  | 0.5 |  |  |  | 0.5 | 0.9 |
| **DSM POP 48h** |  |  |  |  |  |  | 0.1 |  | 0.3 | 5.1 |  | 4.9 |  | 10.4 | 0.4 |
| **DSM POP 96h** |  |  |  | 0.2 |  | 0.3 | 0.1 |  | 0.4 | 6.0 | 1.3 | 6.0 |  | 14.3 | 2.3 |
| **PD630 POP 48h** |  |  |  |  |  |  |  |  |  | 3.2 |  | 2.5 |  | 5.7 |  |
| **PD630 POP 96h** |  | 0.6 |  |  |  |  |  |  | 0.0 |  | 3.4 | 3.6 |  | 7.6 | 0.6 |
| **DSM SWG 48h** | 0.1 |  |  |  | 0.1 | 1.4 |  | 0.8 | 0.2 | 12.2 | 0.3 | 18.0 |  | 33.1 | 2.9 |
| **DSM SWG 96h** |  |  | 0.7 |  |  | 0.2 | 0.1 |  | 0.2 | 5.7 |  | 8.0 |  | 15.0 | 1.2 |
| **PD630 SWG 48h** |  |  |  |  |  |  |  |  | 0.1 | 7.8 |  | 9.9 |  | 17.7 | 0.1 |
| **PD630 SWG 96h** |  |  |  | 0.2 |  | 0.2 | 0.2 |  |  | 4.5 |  | 5.8 |  | 11.0 | 0.7 |

**Sugar content**

|  | avg glucose (mg/ml) | std error | avg galactose (mg/ml) | std error | avg xylose (mg/ml) | std error | avg mannose (mg/ml) | std error |
| --- | --- | --- | --- | --- | --- | --- | --- | --- |
| DAP-SSF PINE 0 h | 9.288E-01 | 4.644E-02 | 3.435E-03 | 1.717E-04 | 1.726E-02 | 8.632E-04 | 2.182E-02 | 1.091E-03 |
| DSM 1069 DAP-SSF PINE 96 h | 8.941E-01 | 4.470E-02 | 3.252E-03 | 1.626E-04 | 1.678E-02 | 8.392E-04 | 2.445E-02 | 1.223E-03 |
| PD 630 DAP-SSF PINE 96 h | 8.616E-01 | 4.308E-02 | 2.827E-03 | 1.413E-04 | 1.578E-02 | 7.892E-04 | 2.363E-02 | 1.182E-03 |
| DAP-SSF POP 0 h | 1.797E+00 | 8.986E-02 | 1.953E-02 | 9.766E-04 | 1.085E-02 | 5.424E-04 | 4.202E-02 | 2.101E-03 |
| DSM 1069 DAP-SSF POP 96 h | 1.491E+00 | 7.455E-02 | 6.807E-03 | 3.403E-04 | 1.063E-02 | 5.314E-04 | 3.504E-02 | 1.752E-03 |
| PD 630 DAP-SSF POP 96 h | 1.524E+00 | 7.619E-02 | 1.317E-02 | 6.585E-04 | 1.041E-02 | 5.204E-04 | 3.853E-02 | 1.926E-03 |
| DAP-SSF SWG 0 h | 1.577E+00 | 7.883E-02 | 8.118E-03 | 4.059E-04 | 1.881E-02 | 9.407E-04 | 5.874E-02 | 2.937E-03 |
| DSM 1069 DAP-SSF SWG 96 h | 1.563E+00 | 7.816E-02 | 7.018E-03 | 3.509E-04 | 1.854E-02 | 9.272E-04 | 5.324E-02 | 2.662E-03 |
| PD 630 DAP-SSF SWG 96 h | 1.117E+00 | 5.586E-02 | 5.918E-03 | 2.959E-04 | 1.565E-02 | 7.823E-04 | 2.358E-02 | 1.179E-03 |
