# Supplementary material for: Utilization of simultaneous saccharification and fermentation residues as feedstock for lipid accumulation in Rhodococcus opacus
Source: AMB Express. 2017 Sep 29;7:185. doi: 10.1186/s13568-017-0484-0 (PMC5622019; doi:10.1186/s13568-017-0484-0)
Supplement: Supplementary file 2 — Additional file 2: Figure S1. Summary of sugars present in DAP-SSF residue before and after fermentation with Rhodococcus after acid-hydrolysis. [file 13568_2017_484_MOESM2_ESM.docx]

**Fig. S1** Summary of sugars present in DAP-SSF residue before and after fermentation with *Rhodococcus* after acid-hydrolysis.

**
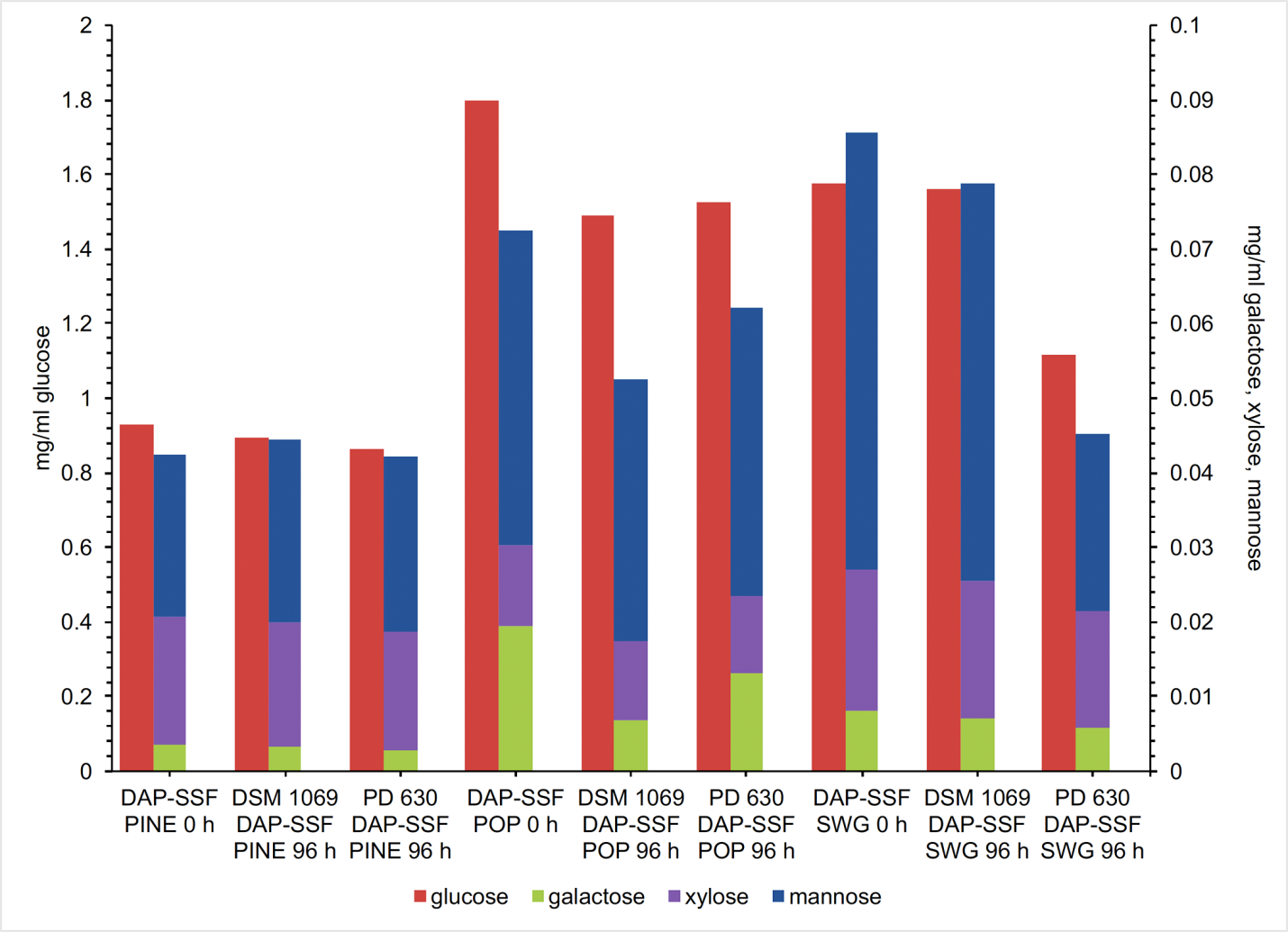
**
