# Supplementary material for: Utilization of simultaneous saccharification and fermentation residues as feedstock for lipid accumulation in Rhodococcus opacus
Source: AMB Express. 2017 Sep 29;7:185. doi: 10.1186/s13568-017-0484-0 (PMC5622019; doi:10.1186/s13568-017-0484-0)
Supplement: Supplementary file 3 — Additional file 3: Figure S2. FTIR spectra of poplar solid residue (obtained after DAP-SSF pretreatment) (a) before and after treatment with two different strains of R. opacus for 96 h, (b) zoomed in fingerprint region of the spectra, and (c) comparison of the relative intensity of the peaks at 1512, 1315 and 1108 cm−1; each spectrum represented is the average of at least two spectra recorded for each sample. [file 13568_2017_484_MOESM3_ESM.docx]

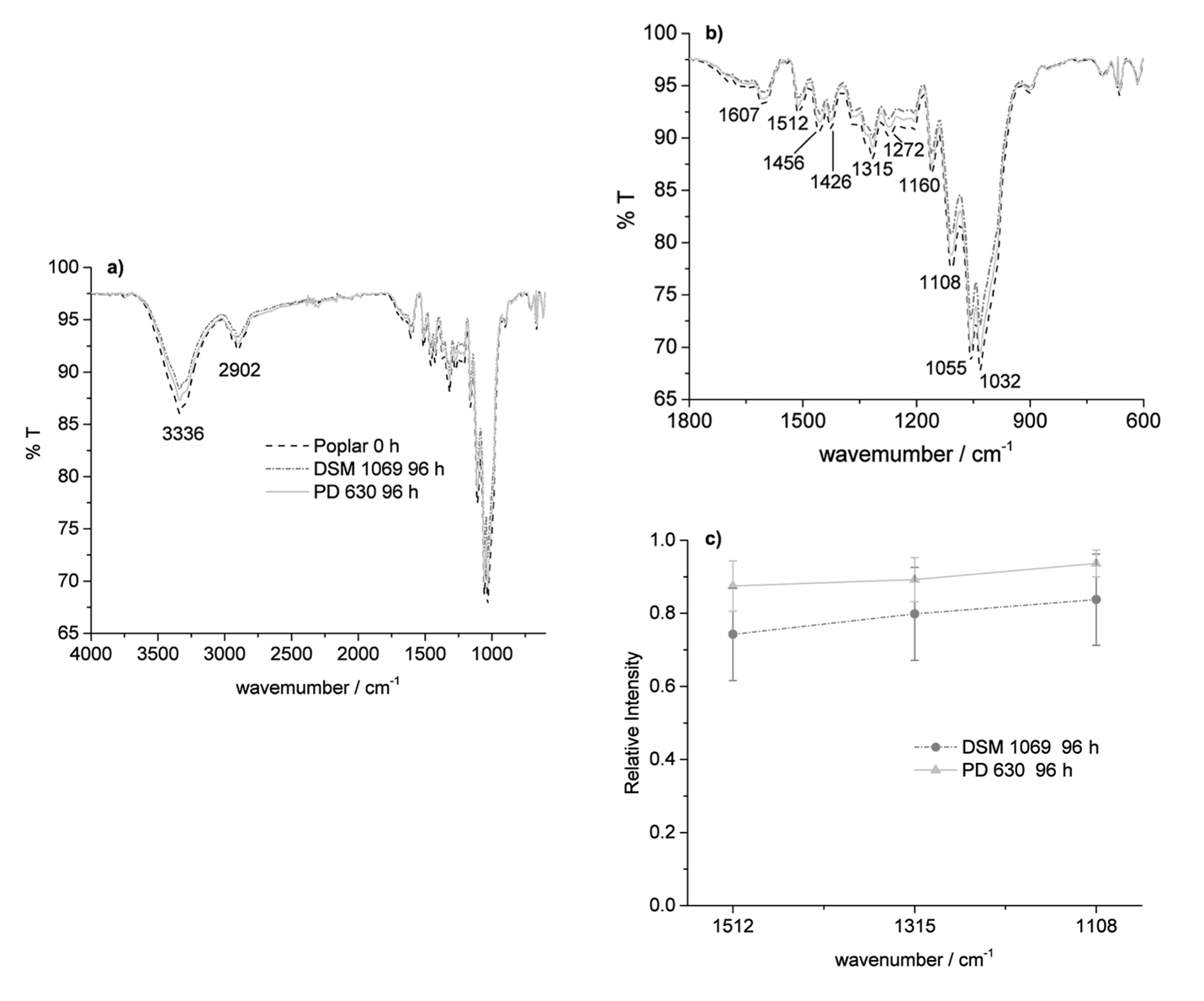


**Fig. S2** a) FTIR spectra of poplar solid residue (obtained after DAP-SSF pretreatment) a) before and after treatment with two different strains of R. opacus for 96 h, (b) zoomed in fingerprint region of the spectra, and c) comparison of the relative intensity of the peaks at 1512, 1315 and 1108 cm^-1^; each spectrum represented is the average of at least two spectra recorded for each sample
