# Supplementary material for: Utilization of simultaneous saccharification and fermentation residues as feedstock for lipid accumulation in Rhodococcus opacus
Source: AMB Express. 2017 Sep 29;7:185. doi: 10.1186/s13568-017-0484-0 (PMC5622019; doi:10.1186/s13568-017-0484-0)
Supplement: Supplementary file 4 — Additional file 4: Figure S3. (a) FTIR spectra of switchgrass residue (obtained after DAP-SSF pretreatment) before and after treatment with two different strains of R. opacus for 96 h, (b) zoomed in fingerprint region of the spectra, and (c) comparison of the relative intensity of the peaks at 1513, 1316 and 1107 cm−1; each spectrum represented is the average of at least two spectra recorded for each sample. [file 13568_2017_484_MOESM4_ESM.docx]

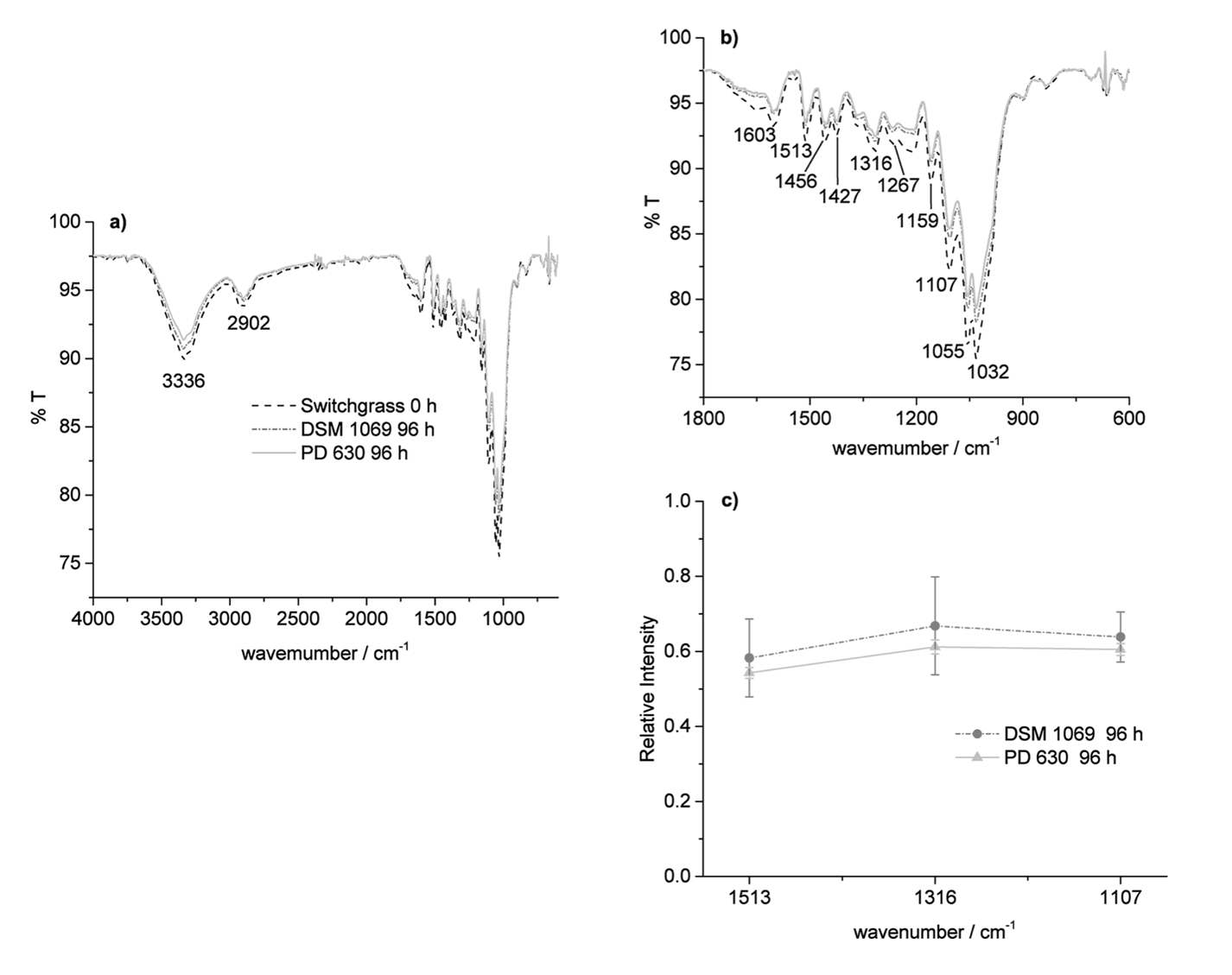


**Fig. S3** FTIR spectra of switchgrass residue (obtained after DAP-SSF pretreatment) before and after treatment with two different strains of R. opacus for 96 h, b) zoomed in fingerprint region of the spectra, and c) comparison of the relative intensity of the peaks at 1513, 1316 and 1107 cm^-1^; each spectrum represented is the average of at least two spectra recorded for each sample
